# A combinatorial optimization approach for diverse motif finding applications

**DOI:** 10.1186/1748-7188-1-13

**Published:** 2006-08-17

**Authors:** Elena Zaslavsky, Mona Singh

**Affiliations:** 1 Department of Computer Science & Lewis-Sigler Institute for Integrative Genomics, Princeton University, Princeton, NJ 08544, USA

## Abstract

**Background:**

Discovering approximately repeated patterns, or motifs, in biological sequences is an important and widely-studied problem in computational molecular biology. Most frequently, motif finding applications arise when identifying shared regulatory signals within DNA sequences or shared functional and structural elements within protein sequences. Due to the diversity of contexts in which motif finding is applied, several variations of the problem are commonly studied.

**Results:**

We introduce a versatile combinatorial optimization framework for motif finding that couples graph pruning techniques with a novel integer linear programming formulation. Our approach is flexible and robust enough to model several variants of the motif finding problem, including those incorporating substitution matrices and phylogenetic distances. Additionally, we give an approach for determining statistical significance of uncovered motifs. In testing on numerous DNA and protein datasets, we demonstrate that our approach typically identifies statistically significant motifs corresponding to either known motifs or other motifs of high conservation. Moreover, in most cases, our approach finds provably optimal solutions to the underlying optimization problem.

**Conclusion:**

Our results demonstrate that a combined graph theoretic and mathematical programming approach can be the basis for effective and powerful techniques for diverse motif finding applications.

## Background

Motif discovery is the problem of finding approximately repeated patterns in unaligned sequence data. It is important in uncovering transcriptional networks, as short common subsequences in genomic data may correspond to a regulatory protein's binding sites, and in protein function identification, where short blocks of conserved amino acids code for important structural or functional elements.

The biological problems addressed by motif finding are complex and varied, and no single currently existing method can solve them completely (e.g., see [1,2]). For DNA sequences, motif finding is often applied to sets of sequences from a single genome that have been identified as possessing a common motif, either through DNA microarray studies [3], ChIP-chip experiments [4] or protein binding microarrays [5]. An orthogonal approach, which attempts to identify regulatory sites among a set of orthologous genes across genomes of varying phylogenetic distance, is adopted by [6-10]. For protein sequences, and especially in the case of divergent sequence motifs, it is particularly useful to incorporate amino acid substitution matrices [11,12]. Often, motif finding methods are either tailor-made to a specific variant of the motif finding problem, or perform very differently when presented with a diverse set of instances.

Numerous approaches to motif finding have been suggested (e.g., [13-24], and those referenced in [1]). These methods differ mainly in the choice of the motif representation, the objective function used for assessing the quality of a motif, and the search procedure used for finding an optimal (or sub-optimal but reasonable) solution. Two broad categories of motif finding algorithms can be identified [25]: stochastic-search methods based on the position-specific scoring matrix (PSSM) representation and combinatorial approaches based on variants of the consensus sequence representation. Both categories come with their own sets of scoring functions (e.g., see [25,26]), and most variants of the motif finding problem are NP-hard, including those optimizing either the average information content score or the sum-of-pairs score [27]. The performance of these two broad groups of methods seem to be complementary in many cases, with a slight performance advantage demonstrated by representative methods of the combinatorial class (e.g., Weeder [24]), as reported in a recent comprehensive study [1]. However, many combinatorial methods enumerate every possible pattern, and are thus limited in the length of the motifs they can search for. While this may be less of an issue in eukaryotic genomes, where transcriptional regulation is mediated combinatorially with a large number of transcription factors with relatively short binding sites, substantially longer motifs are found when considering either DNA binding sites in prokaryotic genomes (e.g., for helix-turn-helix binding domains of transcription factors) or protein motifs [28,29].

Here, we introduce a combinatorial optimization framework for motif finding that is flexible enough to model several variants of the problem and is not limited by the motif length. Underlying our approach, we consider motif discovery as the problem of finding the best gapless local multiple sequence alignment using the sum-of-pairs (SP) scoring scheme. The SP-score is one of many reasonable schemes for assessing motif conservation [30,31]. In the case of motif search, where the goal is to use a set of known motif instances and uncover additional instances, the SP-score has been shown empirically to be comparable to PSSM-based methods [32]. Additionally, unlike the PSSM models, which typically assume independence of motif positions, the SP-score can address the problem of nucleotide or amino acid dependencies in a natural way. This is an important consideration; for example in the case of nucleotides, it has been shown that there are interdependent effects between bases [33,34]. Moreover, modeling these dependencies using the SP-score leads to improved performance in representing and searching for binding sites; a similar statistically significant improvement is not observed when extending PSSMs to incorporate pairwise dependencies [32]. The SP-score was most recently utilized in the context of motif finding in MaMF [13].

In this paper, we use the SP-score and recast the motif finding problem as that of finding a maximum (or minimum) weight clique in a multi-partite graph, and introduce a two-pronged approach, based on graph pruning and mathematical programming, to solve it. In particular, we first formulate the problem as an integer linear program (ILP) and then consider its linear programming relaxation. In practice, the linear programs (LPs) arising from motif finding applications can be prohibitively large, numbering in the millions of variables. Thus, to reduce the size of the LPs, we develop a number of new pruning techniques, building upon the ideas of [35,36]. These fall into the broad category of dead-end elimination (DEE) algorithms (e.g., [37]), where sequence positions that are incompatible with the optimal alignment are discarded. In practice, such methods are very effective in reducing problem size; to handle the rare cases where the DEE techniques do not sufficiently prune the problem instance, we also develop a heuristic iterative scheme to eliminate sequence positions. The reduced linear programs are then solved by the ILOG CPLEX LP solver, and in cases where fractional solutions are found, an ILP solver is applied.

Given a motif discovered by any method, it is important to be able to assess its statistical significance, as even optimal solutions for their respective objective functions may result in very poor motifs. We demonstrate how to test the statistical significance of the motifs discovered via the graph pruning/mathematical programming approach by using the background frequencies for each base or amino acid and computing the motif scores' probability distribution [38]. We then assess the number of motifs of the same or better quality that are expected to occur in the data at random. In the few cases where the heuristic DEE procedure is applied, we are able to give a lower bound on the significance value of the optimal solution; this allows us to evaluate how much better an alternate undiscovered motif might be.

We test our coupled mathematical programming and pruning approach, LP/DEE, in diverse settings. First, we consider the problem of finding shared motifs in protein sequences. Unlike commonly-used PSSM-based methods for motif finding (e.g., [15,18]), our combinatorial formulation naturally incorporates amino acid substitution matrices. For all tested datasets, we find the actual protein motifs exactly, and these motifs correspond to optimal solutions according to the SP scoring scheme. Second, we consider sets of genes known to be regulated by the same *E. coli *transcription factor, and apply our approach to find the corresponding binding sites in genomic sequence data. We compare our results with those of three popular methods [18,22,39], and show that our method is often able to better locate the actual binding sites. Using the same dataset, we also embed *E. coli *binding sites within sequences of increasingly biased composition, and show that our scoring scheme and motif finding procedure is effective in this scenario as well. Third, we consider the phylogenetic footprinting problem [9], and find shared motifs upstream of orthologous genes. The difficulty of this problem lies in that the sequences may not have had enough evolutionary time to diverge and may share sequence level similarity beyond the functionally important site; incorporation of additional information, in the form of weightings obtained from a phylogenetic tree relating the species, proves useful in this context. Finally, we demonstrate in the context of phylogenetic footprinting that our formulation can be used to find multiple solutions, corresponding to several distinct motifs. In all scenarios, we test the uncovered motifs for statistical significance. We show that our method works well in practice, typically recovering statistically significant motifs that correspond to either known motifs or other motifs of high conservation.

Interestingly, the vast majority of motif finding instances considered are not only effectively pruned by the optimality-preserving DEE methods, but also lead to linear programs whose optimal solutions are integral. These two conditions together guarantee optimality of the final solution for the original SP-based motif finding problem. This is interesting, since the motif finding formulation is known to be NP-hard [27], and nevertheless our approach runs in polynomial time for many practical instances of the problem. Overall, the ability of our LP/DEE method to find optimal solutions to large problems demonstrates the power of the computational search procedures, and its performance in uncovering known motifs illustrates its utility for novel sequence motif discovery.

## Methods

### Broad problem formulation

Motif discovery is modeled here as the problem of finding an ungapped local multiple sequence alignment (MSA) of fixed length with the best sum-of-pairs (SP) score. That is, given *N *sequences {*S*_1_, ..., *S*_*N*_} and a block length parameter *l*, the goal is to find an *l*-long subsequence from each input sequence so that the total similarity among selected blocks is maximized. More formally, let sik
 MathType@MTEF@5@5@+=feaafiart1ev1aaatCvAUfKttLearuWrP9MDH5MBPbIqV92AaeXatLxBI9gBaebbnrfifHhDYfgasaacH8akY=wiFfYdH8Gipec8Eeeu0xXdbba9frFj0=OqFfea0dXdd9vqai=hGuQ8kuc9pgc9s8qqaq=dirpe0xb9q8qiLsFr0=vr0=vr0dc8meaabaqaciaacaGaaeqabaqabeGadaaakeaacqWGZbWCdaqhaaWcbaGaemyAaKgabaGaem4AaSgaaaaa@3102@ refer to the *l*-long subsequence in sequence *S*_*i *_beginning in position *k *and let *sim*(*x*, *y*) denote a similarity score between the *l*-long subsequences *x*, *y*. The objective is then to find the set of positions {*k*_1_, ..., *k*_*N*_} in each sequence, such that the SP-score ∑_*i*<*j *_*sim *(siki
 MathType@MTEF@5@5@+=feaafiart1ev1aaatCvAUfKttLearuWrP9MDH5MBPbIqV92AaeXatLxBI9gBaebbnrfifHhDYfgasaacH8akY=wiFfYdH8Gipec8Eeeu0xXdbba9frFj0=OqFfea0dXdd9vqai=hGuQ8kuc9pgc9s8qqaq=dirpe0xb9q8qiLsFr0=vr0=vr0dc8meaabaqaciaacaGaaeqabaqabeGadaaakeaacqWGZbWCdaqhaaWcbaGaemyAaKgabaGaem4AaS2aaSbaaWqaaiabdMgaPbqabaaaaaaa@328A@, sjkj
 MathType@MTEF@5@5@+=feaafiart1ev1aaatCvAUfKttLearuWrP9MDH5MBPbIqV92AaeXatLxBI9gBaebbnrfifHhDYfgasaacH8akY=wiFfYdH8Gipec8Eeeu0xXdbba9frFj0=OqFfea0dXdd9vqai=hGuQ8kuc9pgc9s8qqaq=dirpe0xb9q8qiLsFr0=vr0=vr0dc8meaabaqaciaacaGaaeqabaqabeGadaaakeaacqWGZbWCdaqhaaWcbaGaemOAaOgabaGaem4AaS2aaSbaaWqaaiabdQgaQbqabaaaaaaa@328E@) is maximized.

This problem can be formulated in graph-theoretic terms [40]. Let *G *be an undirected *N*-partite graph with node set *V*_1 _∪ ... ∪ *V*_*N*_, where V_*i *_includes a node *u *for each *l*-long subsequence sik
 MathType@MTEF@5@5@+=feaafiart1ev1aaatCvAUfKttLearuWrP9MDH5MBPbIqV92AaeXatLxBI9gBaebbnrfifHhDYfgasaacH8akY=wiFfYdH8Gipec8Eeeu0xXdbba9frFj0=OqFfea0dXdd9vqai=hGuQ8kuc9pgc9s8qqaq=dirpe0xb9q8qiLsFr0=vr0=vr0dc8meaabaqaciaacaGaaeqabaqabeGadaaakeaacqWGZbWCdaqhaaWcbaGaemyAaKgabaGaem4AaSgaaaaa@3102@ in the *i*-th sequence. Note that the subsequences corresponding to two consecutive vertices overlap in *l *- 1 positions, and that the *V*_*i*_'s may have varying sizes. Each pair of nodes *u *∈ *V*_*i *_and *v *∈ *V*_*j *_(*i *≠ *j*), corresponding to subsequences sik
 MathType@MTEF@5@5@+=feaafiart1ev1aaatCvAUfKttLearuWrP9MDH5MBPbIqV92AaeXatLxBI9gBaebbnrfifHhDYfgasaacH8akY=wiFfYdH8Gipec8Eeeu0xXdbba9frFj0=OqFfea0dXdd9vqai=hGuQ8kuc9pgc9s8qqaq=dirpe0xb9q8qiLsFr0=vr0=vr0dc8meaabaqaciaacaGaaeqabaqabeGadaaakeaacqWGZbWCdaqhaaWcbaGaemyAaKgabaGaem4AaSgaaaaa@3102@ and sjk′
 MathType@MTEF@5@5@+=feaafiart1ev1aaatCvAUfKttLearuWrP9MDH5MBPbIqV92AaeXatLxBI9gBaebbnrfifHhDYfgasaacH8akY=wiFfYdH8Gipec8Eeeu0xXdbba9frFj0=OqFfea0dXdd9vqai=hGuQ8kuc9pgc9s8qqaq=dirpe0xb9q8qiLsFr0=vr0=vr0dc8meaabaqaciaacaGaaeqabaqabeGadaaakeaacqWGZbWCdaqhaaWcbaGaemOAaOgabaGafm4AaSMbauaaaaaaaa@3110@ in *S*_*i *_and *S*_*j *_respectively, is joined by an edge with weight of *w*_*uv *_= *sim *(sik
 MathType@MTEF@5@5@+=feaafiart1ev1aaatCvAUfKttLearuWrP9MDH5MBPbIqV92AaeXatLxBI9gBaebbnrfifHhDYfgasaacH8akY=wiFfYdH8Gipec8Eeeu0xXdbba9frFj0=OqFfea0dXdd9vqai=hGuQ8kuc9pgc9s8qqaq=dirpe0xb9q8qiLsFr0=vr0=vr0dc8meaabaqaciaacaGaaeqabaqabeGadaaakeaacqWGZbWCdaqhaaWcbaGaemyAaKgabaGaem4AaSgaaaaa@3102@, sjk′
 MathType@MTEF@5@5@+=feaafiart1ev1aaatCvAUfKttLearuWrP9MDH5MBPbIqV92AaeXatLxBI9gBaebbnrfifHhDYfgasaacH8akY=wiFfYdH8Gipec8Eeeu0xXdbba9frFj0=OqFfea0dXdd9vqai=hGuQ8kuc9pgc9s8qqaq=dirpe0xb9q8qiLsFr0=vr0=vr0dc8meaabaqaciaacaGaaeqabaqabeGadaaakeaacqWGZbWCdaqhaaWcbaGaemOAaOgabaGafm4AaSMbauaaaaaaaa@3110@). By this construction *G *is a complete *N*-partite graph. The MSA is achieved by picking the highest weight *N*-partite clique in graph *G*.

Similarity between *l*-long subsequences can be defined using a simple scoring scheme, such as counting up the number of matching bases or amino acids when the subsequences are aligned. However, for DNA sequences, the background distribution of the input sequence can vary substantially, and in order to reward matches of more infrequent bases, instead of using 1 for a match, we assign a score of *log*(1/*f*(*b*)) for a base *b *pairing, where *f*(*b*) is the zero-corrected frequency of base *b *in the background, and 0 for any mismatch. (We also experimented with a scheme that assigns a score of 1/*f*(*b*) for a base *b *match; both methods perform similarly). In practice, we work with integral scores by scaling the floating point numbers to the desired degree of accuracy and rounding (here, we use the scale factor of 100). For protein sequences, on the other hand, compositional bias is not as major an issue, and instead, to better capture the relationships between the amino acids, we score the similarity between two amino acids using substitution matrices. This assigns higher scores to more favorable substitutions and better reflects shared biochemical properties of such pairings. We experiment with both PAM [11] and BLOSUM [12] matrix families.

### Integer linear programming formulation

The motif finding problem can be formulated as an ILP as follows. For a graph *G *= (*V*, *E*) corresponding to the motif finding problem, where *V *= *V*_1 _∪ ... ∪ *V*_*N *_and *E *= {(*u*, *v*): *u *∈ *V*_*i*_, *v *∈ *V*_*j*_, *i *≠ *j*}, we introduce a binary decision variable *x*_*u *_for every vertex *u*, and a binary decision variable *y*_*uv *_for every edge (*u*, *v*). Setting *x*_*u *_to 1 corresponds to selecting vertex *u *for the clique and thus choosing the sequence position corresponding to *u *in the alignment. Edge variable *y*_*uv *_is set to 1 if both endpoints *u *and *v *are selected for the clique. Then the following ILP solves the motif finding problem formulated above:

Maximize∑(u,v)∈Ewuv⋅yuvsubject to∑u∈Vjxu=1for 1≤j≤N(node constraints)∑u∈Vjyuv=xvfor 1≤j≤N,v∈V\Vj(edge constraints)xu,yuv∈{0,1}for u∈V,(u,v)∈E
 MathType@MTEF@5@5@+=feaafiart1ev1aaatCvAUfKttLearuWrP9MDH5MBPbIqV92AaeXatLxBI9gBaebbnrfifHhDYfgasaacH8akY=wiFfYdH8Gipec8Eeeu0xXdbba9frFj0=OqFfea0dXdd9vqai=hGuQ8kuc9pgc9s8qqaq=dirpe0xb9q8qiLsFr0=vr0=vr0dc8meaabaqaciaacaGaaeqabaqabeGadaaakeaafaqaaeqbdaaaaeaacqqGnbqtcqqGHbqycqqG4baEcqqGPbqAcqqGTbqBcqqGPbqAcqqG6bGEcqqGLbqzaeaadaaeqaqaaiabdEha3naaBaaaleaacqWG1bqDcqWG2bGDaeqaaOGaeyyXICTaemyEaK3aaSbaaSqaaiabdwha1jabdAha2bqabaaabaGaeiikaGIaemyDauNaeiilaWIaemODayNaeiykaKIaeyicI4SaemyraueabeqdcqGHris5aaGcbaaabaGaee4CamNaeeyDauNaeeOyaiMaeeOAaOMaeeyzauMaee4yamMaeeiDaqNaeeiiaaIaeeiDaqNaee4Ba8gabaaabaaabaWaaabeaeaacqWG4baEdaWgaaWcbaGaemyDauhabeaakiabg2da9iabigdaXaWcbaGaemyDauNaeyicI4SaemOvay1aaSbaaWqaaiabdQgaQbqabaaaleqaniabggHiLdaakeaacqqGMbGzcqqGVbWBcqqGYbGCcqqGGaaicqaIXaqmcqGHKjYOcqWGQbGAcqGHKjYOcqWGobGtaeaacqGGOaakcqWGUbGBcqWGVbWBcqWGKbazcqWGLbqzcqqGGaaicqqGJbWycqqGVbWBcqqGUbGBcqqGZbWCcqqG0baDcqqGYbGCcqqGHbqycqqGPbqAcqqGUbGBcqqG0baDcqqGZbWCcqGGPaqkaeaadaaeqaqaaiabdMha5naaBaaaleaacqWG1bqDcqWG2bGDaeqaaOGaeyypa0JaemiEaG3aaSbaaSqaaiabdAha2bqabaaabaGaemyDauNaeyicI4SaemOvay1aaSbaaWqaaiabdQgaQbqabaaaleqaniabggHiLdaakeaacqqGMbGzcqqGVbWBcqqGYbGCcqqGGaaicqaIXaqmcqGHKjYOcqWGQbGAcqGHKjYOcqWGobGtcqGGSaalcqWG2bGDcqGHiiIZcqWGwbGvcqGGCbaxcqWGwbGvdaWgaaWcbaGaemOAaOgabeaaaOqaaiabcIcaOiabdwgaLjabdsgaKjabdEgaNjabdwgaLjabbccaGiabbogaJjabb+gaVjabb6gaUjabbohaZjabbsha0jabbkhaYjabbggaHjabbMgaPjabb6gaUjabbsha0jabbohaZjabcMcaPaqaaiabdIha4naaBaaaleaacqWG1bqDaeqaaOGaeiilaWIaemyEaK3aaSbaaSqaaiabdwha1jabdAha2bqabaGccqGHiiIZcqGG7bWEcqaIWaamcqGGSaalcqaIXaqmcqGG9bqFaeaacqqGMbGzcqqGVbWBcqqGYbGCcqqGGaaicqWG1bqDcqGHiiIZcqWGwbGvcqGGSaalcqGGOaakcqWG1bqDcqGGSaalcqWG2bGDcqGGPaqkcqGHiiIZcqWGfbqraeaaaaaaaa@E7E9@

The first set of constraints ensures that exactly one vertex is picked from every graph part, corresponding to one position being chosen from every input sequence. The second set of constraints relates vertex variables to edge variables, allowing the objective function to be expressed in terms of finding a maximum edge-weight clique. An edge is chosen only if it connects two chosen vertices. This formulation is similar to that used by us [41] in fixed-backbone protein design and homology modeling.

ILP itself is NP-hard, but replacing the integrality constraints on the *x *and *y *variables with 0 ≤ *x*_*u*_, *y*_*uv *_≤ 1 gives an LP that can be solved in polynomial time. If the LP solution happens to be integral, it is guaranteed to be optimal for the original ILP and motif finding problem. Non-integral solutions, on the other hand, are not feasible for the ILP and do not translate to a selection of positions for the MSA problem; in these cases, more computationally intensive ILP solvers must be invoked.

### Graph pruning techniques

In this section, we introduce a number of successively more powerful optimality-preserving dead-end elimination (DEE) techniques for pruning graphs corresponding to motif finding problems. The basic idea is to discard vertices and/or edges that cannot possibly be part of the optimal solution.

#### Basic clique-bounds DEE

The idea of our first pruning technique is as follows. Suppose there exists a clique of weight C* in *G*. Then a vertex *u*, whose participation in any possible clique in *G *reduces the weight of that clique below C*, is incompatible with the optimal alignment and can be safely eliminated (similar to [36]).

For *u *∈ *V*_*i *_define *star*(*u*) to be a selection of vertices from every graph part other than *V*_*i*_. Let *F*_*u *_be the value induced by the edge weights for a *star*(*u*) that form best pairwise alignments with *u*:

Fu=∑j≠imax⁡v∈Vj wuv     (1)
 MathType@MTEF@5@5@+=feaafiart1ev1aaatCvAUfKttLearuWrP9MDH5MBPbIqV92AaeXatLxBI9gBaebbnrfifHhDYfgasaacH8akY=wiFfYdH8Gipec8Eeeu0xXdbba9frFj0=OqFfea0dXdd9vqai=hGuQ8kuc9pgc9s8qqaq=dirpe0xb9q8qiLsFr0=vr0=vr0dc8meaabaqaciaacaGaaeqabaqabeGadaaakeaacqWGgbGrdaWgaaWcbaGaemyDauhabeaakiabg2da9maaqafabaWaaCbeaeaacyGGTbqBcqGGHbqycqGG4baEaSqaaiabdAha2jabgIGiolabdAfawnaaBaaameaacqWGQbGAaeqaaaWcbeaakiabbccaGaWcbaGaemOAaOMaeyiyIKRaemyAaKgabeqdcqGHris5aOGaem4DaC3aaSbaaSqaaiabdwha1jabdAha2bqabaGccaWLjaGaaCzcamaabmGabaGaeGymaedacaGLOaGaayzkaaaaaa@4A63@

If *u *were to participate in any clique in *G*, it cannot possibly contribute more than *F*_*u *_to the weight of the clique. Similarly, let  be the value of the best possible *star*(*u*) among all *u *∈ *V*_*i*_:

Fi∗=max⁡u∈ViFu     (2)
 MathType@MTEF@5@5@+=feaafiart1ev1aaatCvAUfKttLearuWrP9MDH5MBPbIqV92AaeXatLxBI9gBaebbnrfifHhDYfgasaacH8akY=wiFfYdH8Gipec8Eeeu0xXdbba9frFj0=OqFfea0dXdd9vqai=hGuQ8kuc9pgc9s8qqaq=dirpe0xb9q8qiLsFr0=vr0=vr0dc8meaabaqaciaacaGaaeqabaqabeGadaaakeaacqWGgbGrdaqhaaWcbaGaemyAaKgabaGaey4fIOcaaOGaeyypa0ZaaCbeaeaacyGGTbqBcqGGHbqycqGG4baEaSqaaiabdwha1jabgIGiolabdAfawnaaBaaameaacqWGPbqAaeqaaaWcbeaakiabdAeagnaaBaaaleaacqWG1bqDaeqaaOGaaCzcaiaaxMaadaqadiqaaiabikdaYaGaayjkaiaawMcaaaaa@41EF@

Fi∗
 MathType@MTEF@5@5@+=feaafiart1ev1aaatCvAUfKttLearuWrP9MDH5MBPbIqV92AaeXatLxBI9gBaebbnrfifHhDYfgasaacH8akY=wiFfYdH8Gipec8Eeeu0xXdbba9frFj0=OqFfea0dXdd9vqai=hGuQ8kuc9pgc9s8qqaq=dirpe0xb9q8qiLsFr0=vr0=vr0dc8meaabaqaciaacaGaaeqabaqabeGadaaakeaacqWGgbGrdaqhaaWcbaGaemyAaKgabaGaey4fIOcaaaaa@3038@ is an upper bound on what any vertex in *V*_*i *_can contribute to any alignment.

Now, if *F*_*z*_, the most a vertex *z *∈ *V*_*k *_can contribute to a clique, assuming the best possible contributions from all other graph parts, is insufficient compared to the value C* of an existing clique, i.e. if

Fz<2×C∗−∑i≠kFi∗,     (3)
 MathType@MTEF@5@5@+=feaafiart1ev1aaatCvAUfKttLearuWrP9MDH5MBPbIqV92AaeXatLxBI9gBaebbnrfifHhDYfgasaacH8akY=wiFfYdH8Gipec8Eeeu0xXdbba9frFj0=OqFfea0dXdd9vqai=hGuQ8kuc9pgc9s8qqaq=dirpe0xb9q8qiLsFr0=vr0=vr0dc8meaabaqaciaacaGaaeqabaqabeGadaaakeaacqWGgbGrdaWgaaWcbaGaemOEaOhabeaakiabgYda8iabikdaYiabgEna0kabdoeadnaaCaaaleqabaGaey4fIOcaaOGaeyOeI0YaaabuaeaacqWGgbGrdaqhaaWcbaGaemyAaKgabaGaey4fIOcaaaqaaiabdMgaPjabgcMi5kabdUgaRbqab0GaeyyeIuoakiabcYcaSiaaxMaacaWLjaWaaeWaceaacqaIZaWmaiaawIcacaGLPaaaaaa@4575@

*z *can be discarded. The clique value C* is used with a factor of 2 since two edges are accounted for between every pair of graph parts in the above inequality.

In fact, the values of Fi∗
 MathType@MTEF@5@5@+=feaafiart1ev1aaatCvAUfKttLearuWrP9MDH5MBPbIqV92AaeXatLxBI9gBaebbnrfifHhDYfgasaacH8akY=wiFfYdH8Gipec8Eeeu0xXdbba9frFj0=OqFfea0dXdd9vqai=hGuQ8kuc9pgc9s8qqaq=dirpe0xb9q8qiLsFr0=vr0=vr0dc8meaabaqaciaacaGaaeqabaqabeGadaaakeaacqWGgbGrdaqhaaWcbaGaemyAaKgabaGaey4fIOcaaaaa@3038@ are further constrained by requiring a connection to *z *when *z *is under consideration. That is, when considering a node *z *∈ *V*_*k *_to eliminate, and calculating Fi∗
 MathType@MTEF@5@5@+=feaafiart1ev1aaatCvAUfKttLearuWrP9MDH5MBPbIqV92AaeXatLxBI9gBaebbnrfifHhDYfgasaacH8akY=wiFfYdH8Gipec8Eeeu0xXdbba9frFj0=OqFfea0dXdd9vqai=hGuQ8kuc9pgc9s8qqaq=dirpe0xb9q8qiLsFr0=vr0=vr0dc8meaabaqaciaacaGaaeqabaqabeGadaaakeaacqWGgbGrdaqhaaWcbaGaemyAaKgabaGaey4fIOcaaaaa@3038@ according to Equation 2 among all possible *u *∈ *V*_*i*_, the *F*_*u *_of Equation 1 is instead computed as:

Fu=wzu+∑j≠i,kmax⁡v∈Vj wuv     (4)
 MathType@MTEF@5@5@+=feaafiart1ev1aaatCvAUfKttLearuWrP9MDH5MBPbIqV92AaeXatLxBI9gBaebbnrfifHhDYfgasaacH8akY=wiFfYdH8Gipec8Eeeu0xXdbba9frFj0=OqFfea0dXdd9vqai=hGuQ8kuc9pgc9s8qqaq=dirpe0xb9q8qiLsFr0=vr0=vr0dc8meaabaqaciaacaGaaeqabaqabeGadaaakeaacqWGgbGrdaWgaaWcbaGaemyDauhabeaakiabg2da9iabdEha3naaBaaaleaacqWG6bGEcqWG1bqDaeqaaOGaey4kaSYaaabuaeaadaWfqaqaaiGbc2gaTjabcggaHjabcIha4bWcbaGaemODayNaeyicI4SaemOvay1aaSbaaWqaaiabdQgaQbqabaaaleqaaaqaaiabdQgaQjabgcMi5kabdMgaPjabcYcaSiabdUgaRbqab0GaeyyeIuoakiabbccaGiabdEha3naaBaaaleaacqWG1bqDcqWG2bGDaeqaaOGaaCzcaiaaxMaadaqadiqaaiabisda0aGaayjkaiaawMcaaaaa@5212@

The value of C* can be computed from any "good" alignment. We use the weight of the clique imposed by the best overall *star*.

#### Tighter constraints for clique-bounds DEE

For a vertex *u *∈ *V*_*i *_and every other *V*_*j*_, an edge has to connect *u *to some *v *∈ *V*_*j *_in any alignment. When calculating *F*_*u*_, we can constrain its value by considering three-way alignments and requiring that the vertices in the best *star*(*u*) chosen as neighbors of *u *in graph parts other than *V*_*j *_are also good matches to *v*. Performing this computation for every pair of *u*, *V*_*j *_and considering every edge incident on *u *would be too costly. Therefore, we only consider such three-way alignments for every vertex *u *∈ *V*_*i *_and the next part *V*_*i*+1 _of the graph (with the last and first parts paired). Essentially, this procedure shifts the emphasis onto edges, allowing better alignments and bounds, and yet eliminates vertices by considering the best edge incident on it.

For a given edge (*u*, *v*) with endpoints *u *∈ *V*_*i *_and *v *∈ *V*_*i*+1 _we consider an adjacent *double star *with two centers at *u *and *v*, and sharing all the endpoints *x*_*j *_in the other graph parts, denoted as *dstar*(*u*, *v*); the weight of such a *dstar*(*u*, *v*) is 2wuv+∑j≠ij≠i+1(wuxj+wvxj)
 MathType@MTEF@5@5@+=feaafiart1ev1aaatCvAUfKttLearuWrP9MDH5MBPbIqV92AaeXatLxBI9gBaebbnrfifHhDYfgasaacH8akY=wiFfYdH8Gipec8Eeeu0xXdbba9frFj0=OqFfea0dXdd9vqai=hGuQ8kuc9pgc9s8qqaq=dirpe0xb9q8qiLsFr0=vr0=vr0dc8meaabaqaciaacaGaaeqabaqabeGadaaakeaacqaIYaGmcqWG3bWDdaWgaaWcbaGaemyDauNaemODayhabeaakiabgUcaRmaaqadabaGaeiikaGIaem4DaC3aaSbaaSqaaiabdwha1jabdIha4naaBaaameaacqWGQbGAaeqaaaWcbeaakiabgUcaRiabdEha3naaBaaaleaacqWG2bGDcqWG4baEdaWgaaadbaGaemOAaOgabeaaaSqabaGccqGGPaqkaSqaauaabeqaceaaaeaacqWGQbGAcqGHGjsUcqWGPbqAaeaacqWGQbGAcqGHGjsUcqWGPbqAcqGHRaWkcqaIXaqmaaaabaaaniabggHiLdaaaa@4EE6@. Now consider a clique {*u*_1 _∈ *V*_1_, ..., *u*_*N *_∈ *V*_*N*_} of some value C*, and the sum of its *double stars:*

∑i=1N(2wuiui+1+∑j≠ij≠i+1(wujui+wujui+1))=2∑i=1N∑j≠iwujui     (5)
 MathType@MTEF@5@5@+=feaafiart1ev1aaatCvAUfKttLearuWrP9MDH5MBPbIqV92AaeXatLxBI9gBaebbnrfifHhDYfgasaacH8akY=wiFfYdH8Gipec8Eeeu0xXdbba9frFj0=OqFfea0dXdd9vqai=hGuQ8kuc9pgc9s8qqaq=dirpe0xb9q8qiLsFr0=vr0=vr0dc8meaabaqaciaacaGaaeqabaqabeGadaaakeaadaaeWbqaaiabcIcaOiabikdaYiabdEha3naaBaaaleaacqWG1bqDdaWgaaadbaGaemyAaKgabeaaliabdwha1naaBaaameaacqWGPbqAcqGHRaWkcqaIXaqmaeqaaaWcbeaakiabgUcaRmaaqafabaGaeiikaGIaem4DaC3aaSbaaSqaaiabdwha1naaBaaameaacqWGQbGAaeqaaSGaemyDau3aaSbaaWqaaiabdMgaPbqabaaaleqaaOGaey4kaSIaem4DaC3aaSbaaSqaaiabdwha1naaBaaameaacqWGQbGAaeqaaSGaemyDau3aaSbaaWqaaiabdMgaPjabgUcaRiabigdaXaqabaaaleqaaOGaeiykaKIaeiykaKcaleaafaqabeGabaaabaGaemOAaOMaeyiyIKRaemyAaKgabaGaemOAaOMaeyiyIKRaemyAaKMaey4kaSIaeGymaedaaaqab0GaeyyeIuoaaSqaaiabdMgaPjabg2da9iabigdaXaqaaiabd6eaobqdcqGHris5aOGaeyypa0JaeGOmaiZaaabCaeaadaaeqbqaaiabdEha3naaBaaaleaacqWG1bqDdaWgaaadbaGaemOAaOgabeaaliabdwha1naaBaaameaacqWGPbqAaeqaaaWcbeaaaeaacqWGQbGAcqGHGjsUcqWGPbqAaeqaniabggHiLdaaleaacqWGPbqAcqGH9aqpcqaIXaqmaeaacqWGobGta0GaeyyeIuoakiaaxMaacaWLjaWaaeWaceaacqaI1aqnaiaawIcacaGLPaaaaaa@7C24@

This sum is equal to 4C*, as each edge (*u*_*i*_, *u*_*j*_) is counted four times. We define *F*_*uv *_with for an edge (*u*, *v*) with endpoints *u *∈ *V*_*i *_and *v *∈ *V*_*i*+1 _as



*F*_*uv *_can be viewed as the weight of the best *dstar *centered at the pair of vertices *u*, *v *(or edge (*u*, *v*)) and it is the best possible contribution to any alignment, if the edge (*u*, *v*) was required to be a part of the alignment. We define *F*_*u *_for *u *∈ *V*_*i *_and Fi∗
 MathType@MTEF@5@5@+=feaafiart1ev1aaatCvAUfKttLearuWrP9MDH5MBPbIqV92AaeXatLxBI9gBaebbnrfifHhDYfgasaacH8akY=wiFfYdH8Gipec8Eeeu0xXdbba9frFj0=OqFfea0dXdd9vqai=hGuQ8kuc9pgc9s8qqaq=dirpe0xb9q8qiLsFr0=vr0=vr0dc8meaabaqaciaacaGaaeqabaqabeGadaaakeaacqWGgbGrdaqhaaWcbaGaemyAaKgabaGaey4fIOcaaaaa@3038@ for part *i *similarly to the above definitions as

Fu=max⁡v∈Vi+1Fuv     (7)
 MathType@MTEF@5@5@+=feaafiart1ev1aaatCvAUfKttLearuWrP9MDH5MBPbIqV92AaeXatLxBI9gBaebbnrfifHhDYfgasaacH8akY=wiFfYdH8Gipec8Eeeu0xXdbba9frFj0=OqFfea0dXdd9vqai=hGuQ8kuc9pgc9s8qqaq=dirpe0xb9q8qiLsFr0=vr0=vr0dc8meaabaqaciaacaGaaeqabaqabeGadaaakeaacqWGgbGrdaWgaaWcbaGaemyDauhabeaakiabg2da9maaxababaGagiyBa0MaeiyyaeMaeiiEaGhaleaacqWG2bGDcqGHiiIZcqWGwbGvdaWgaaadbaGaemyAaKMaey4kaSIaeGymaedabeaaaSqabaGccqWGgbGrdaWgaaWcbaGaemyDauNaemODayhabeaakiaaxMaacaWLjaWaaeWaceaacqaI3aWnaiaawIcacaGLPaaaaaa@446A@

Fi∗=max⁡u∈ViFu     (8)
 MathType@MTEF@5@5@+=feaafiart1ev1aaatCvAUfKttLearuWrP9MDH5MBPbIqV92AaeXatLxBI9gBaebbnrfifHhDYfgasaacH8akY=wiFfYdH8Gipec8Eeeu0xXdbba9frFj0=OqFfea0dXdd9vqai=hGuQ8kuc9pgc9s8qqaq=dirpe0xb9q8qiLsFr0=vr0=vr0dc8meaabaqaciaacaGaaeqabaqabeGadaaakeaacqWGgbGrdaqhaaWcbaGaemyAaKgabaGaey4fIOcaaOGaeyypa0ZaaCbeaeaacyGGTbqBcqGGHbqycqGG4baEaSqaaiabdwha1jabgIGiolabdAfawnaaBaaameaacqWGPbqAaeqaaaWcbeaakiabdAeagnaaBaaaleaacqWG1bqDaeqaaOGaaCzcaiaaxMaadaqadiqaaiabiIda4aGaayjkaiaawMcaaaaa@41FB@

*F*_*u *_is the value of the best *dstar *centered on vertex *u *∈ *V*_*i *_and some vertex *v *∈ *V*_*i*+1_, and Fi∗
 MathType@MTEF@5@5@+=feaafiart1ev1aaatCvAUfKttLearuWrP9MDH5MBPbIqV92AaeXatLxBI9gBaebbnrfifHhDYfgasaacH8akY=wiFfYdH8Gipec8Eeeu0xXdbba9frFj0=OqFfea0dXdd9vqai=hGuQ8kuc9pgc9s8qqaq=dirpe0xb9q8qiLsFr0=vr0=vr0dc8meaabaqaciaacaGaaeqabaqabeGadaaakeaacqWGgbGrdaqhaaWcbaGaemyAaKgabaGaey4fIOcaaaaa@3038@ is the value of the best *dstar *centered on any pair of vertices *u *∈ *V*_*i *_and *v *∈ *V*_*i*+1_.

For any clique {*u*_1 _∈ *V*_1_, ..., *u*_*N *_∈ *V*_*N*_} of value C* in the graph, by Equations 5–8 we have

4C∗≤∑i=1...NFuiui+1≤∑i=1...NFui≤∑i=1...NFi∗     (9)
 MathType@MTEF@5@5@+=feaafiart1ev1aaatCvAUfKttLearuWrP9MDH5MBPbIqV92AaeXatLxBI9gBaebbnrfifHhDYfgasaacH8akY=wiFfYdH8Gipec8Eeeu0xXdbba9frFj0=OqFfea0dXdd9vqai=hGuQ8kuc9pgc9s8qqaq=dirpe0xb9q8qiLsFr0=vr0=vr0dc8meaabaqaciaacaGaaeqabaqabeGadaaakeaacqaI0aancqWGdbWqdaahaaWcbeqaaiabgEHiQaaakiabgsMiJoaaqafabaGaemOray0aaSbaaSqaaiabdwha1naaBaaameaacqWGPbqAaeqaaSGaemyDau3aaSbaaWqaaiabdMgaPjabgUcaRiabigdaXaqabaaaleqaaaqaaiabdMgaPjabg2da9iabigdaXiabc6caUiabc6caUiabc6caUiabd6eaobqab0GaeyyeIuoakiabgsMiJoaaqafabaGaemOray0aaSbaaSqaaiabdwha1naaBaaameaacqWGPbqAaeqaaaWcbeaaaeaacqWGPbqAcqGH9aqpcqaIXaqmcqGGUaGlcqGGUaGlcqGGUaGlcqWGobGtaeqaniabggHiLdGccqGHKjYOdaaeqbqaaiabdAeagnaaDaaaleaacqWGPbqAaeaacqGHxiIkaaaabaGaemyAaKMaeyypa0JaeGymaeJaeiOla4IaeiOla4IaeiOla4IaemOta4eabeqdcqGHris5aOGaaCzcaiaaxMaadaqadiqaaiabiMda5aGaayjkaiaawMcaaaaa@6583@

Then Equation 3, with 2C* replaced by 4C*, can be used to eliminate vertices in the same way as before, eliminating a vertex *z *in a particular graph part if *F*_*z*_, the value of its best adjacent *dstar*, is insufficient considering best possible contributions from all other graph parts. For best pruning results the value of C* should be as high as possible; we choose C* as the clique weight induced by the best overall *dstar*.

#### Graph decomposition

We also use a divide-and-conquer graph decomposition approach for pruning vertices. For every graph part *i *and vertex *u *∈ *V*_*i *_we consider induced subgraphs *G*^*u *^= (*V*^*u*^, *E*^*u*^) in turn, where *V*^*u *^= *u *∪ *V*\*V*_*i*_. Application of the *clique-bounds *DEE technique to graphs *G*^*u *^is very effective since one of the graph parts, Giu
 MathType@MTEF@5@5@+=feaafiart1ev1aaatCvAUfKttLearuWrP9MDH5MBPbIqV92AaeXatLxBI9gBaebbnrfifHhDYfgasaacH8akY=wiFfYdH8Gipec8Eeeu0xXdbba9frFj0=OqFfea0dXdd9vqai=hGuQ8kuc9pgc9s8qqaq=dirpe0xb9q8qiLsFr0=vr0=vr0dc8meaabaqaciaacaGaaeqabaqabeGadaaakeaacqWGhbWrdaqhaaWcbaGaemyAaKgabaGaemyDauhaaaaa@30BE@ contains only one vertex, *u*, and all the *F *and F* values that need to be recomputed for the new graph *G*^*u *^are greatly constrained. The process of updating the *F *and F* values is efficient as the changes are localized to one part in the graph. Importantly, the best known clique value C* remains intact, since the clique of that larger value exists in the original graph and can be used for the decomposed one, helping to eliminate vertices. For some of the vertices *u*, iterative application of the DEE criterion and re-computation of the *F *and F* values causes *G*^*u *^to become disconnected, implying that vertex *u *cannot be part of the optimal alignment. Such a vertex *u *is marked for deletion, and that information is propagated to all subsequently considered induced subgraphs, further constraining the corresponding *F *and F* values and helping to eliminate other vertices in turn.

### Statistical significance

Once we have found a motif of a particular SP-score, we evaluate its statistical significance by calculating the number of motifs of equal or better quality expected to occur in random data with the same characteristics. Let the score of the motif of length *l *in question be denoted by *s*, and let *f*(*b*) be the zero-corrected background frequency of nucleotide (or residue) *b *in the input sequences, and *sim*(*b*_1_, *b*_2_) be the integral score computed for all residue pairs as above. We compute *P*_*l*_(*X*), the probability distribution of scores for a motif of length *l *in *N *sequences, in the first two steps of the following, and infer the e-value of score *s *in the last two:

1. Calculate the exact probability distribution *P*_1_(*X*) for a single column of *N *random residues. We use the multinomial distribution to compute the probability of observing every combination of bases (or residues) in the column according to the background distribution, and calculate the corresponding SP-score for the column. We then add probabilities for the same scores resulting from different base combinations. To make the computation feasible for the protein alphabet and for large numbers of sequences, we calculate the scores and probabilities in such an order that every new score and probability is computable from the previous one by a local update operation.

2. Calculate the probability distribution *P*_*l*_(*X*) for *l *random columns by convolution of *P*_1_(*X*)as in [38], where we inductively construct a distribution for *i *columns based on the distribution for *i *- 1 columns, *P*_*i*-1_(*X*), and the single column distribution *P*_1_(*X*).

3. For a given score *s *of interest, we calculate the probability that an *l*-long pattern has score greater than or equal to *s *by chance alone. This probability is ∑_*x*>=*s *_P_*l*_(*x*).

4. Finally, we compute the total number of possible motifs of length *l *in the data. If the sequences have lengths *L*_1_, ..., *L*_*N*_, then the search space size *L *= ∏_*i *_(*L*_*i *_- *l *+ 1). The expected number of alignments with score at least *s *by chance alone, or the e-value, is equal to L* ∑_*x*>=*s *_P_*l*_(*x*).

### Overview of approach

Our basic LP/DEE approach is to: (1) formulate an instance of motif finding as a graph problem (2) apply the DEE techniques described above in the order of increasing complexity so as to prune the graph (3) use mathematical programming to find a solution to the smaller graph problem and (4) evaluate statistical significance.

While applying DEE, if the size of the graph becomes small enough (set at 800 vertices for the described experiments), we submit the appropriate LP to the CPLEX LP solver and, if necessary, to the ILP solver. To reduce the graph to that necessary small size, we apply the DEE variants, running each one of them until either the specified graph size has been reached, or to convergence so that no further pruning is possible. In particular, we first attempt to prune the graph using *basic clique-bounds DEE*, then we consider tighter bound computations, and lastly we employ *graph decomposition *in conjunction with the DEE methods.

In rare cases the optimality-preserving DEE procedures are unable to prune the graph, and we perform what we call speculative pruning using higher C* values, which do not necessarily correspond to known cliques in graph *G*. Three outcomes of such pruning are possible: (i) The graph is eliminated completely. This guarantees that a clique of value C* does not exist in *G*. (ii) The pruning proves once again insufficient to reduce the graph. (iii) The pruning procedure converges to a small graph. We search the space of possible C* values until we find one that produces outcome (iii). To identify such a value we first translate the possible clique scores into their corresponding e-values, and then perform binary search on the e-value exponent. This method converges quickly, typically locating an appropriate C* in fewer than 10 iterations. If the optimal solution for the final reduced graph is better than the C* used in pruning, then it is also optimal for the original graph. Otherwise, the e-value corresponding to C* provides us with a lower bound on the significance of the actual optimal solution.

### Extensions for other motif finding frameworks

#### Phylogenetic footprinting

An increasingly common way of finding regulatory sites is to look for them among upstream regions of a set of orthologous genes across species (e.g., [9]). In this case additional data, in the form of the phylogenetic tree relating the species, is available and can be exploited. This is especially important when closely related species are part of the input, and, unweighted, they contribute duplicate information and skew the alignment. We use a phylogenetic tree and branch lengths when calculating the edge weights in the graph, with highly diverged sequence pairs getting larger weights. The precise weighting scheme follows the ideas of weighted progressive alignment [42], in which weights *α*_*i *_are computed for every sequence *i*. The calculation sums branch lengths along the path from the tree root to the sequence at the leaf, splitting shared branches among the descendant leaves, and thereby reducing the weight for related sequences. In essence, we solve a multiple sequence alignment problem with weighted SP-score using match/mismatch, where the computed weight for a pair of positions in sequences *i *and *j *is multiplied by *α*_*i *_× *α*_*j*_. The rest of the algorithm operates as in the basic motif finding case above, employing the same LP formulation and DEE techniques.

#### Subtle motifs

Another widely studied formulation of motif finding is the 'subtle' motifs formulation [17], in which an unknown pattern of a length *l *is implanted with *d *modifications into each of the input sequences. The graph version of the problem remains the same except that edges only exist between two vertices that correspond to subsequences whose Hamming distance is at most 2*d *(since otherwise they cannot both be implanted instances of the same pattern). Edges can either be unweighted, or weighted by the number of mismatches between the corresponding subsequences. Either is easily modeled via slight modification of the ILP given earlier (with variables corresponding to non-existent edges removed, and summations in the *edge *constraints taken only over existing edges), and the resulting ILP can be used in conjunction with the numerous graph pruning techniques previously developed for this problem (e.g. [17]).

#### Multiple motifs

Here we give extensions to address the issue of multiple motifs existing in a set of sequences. Discovery of distinct multiple motifs, such as sets of binding sites for two different transcription factors, can be done iteratively by first locating a single optimal motif, masking it out from the problem instance, and then looking for the next one. We mask the previous motif by deleting its solution vertices from the original graph, and then reapplying the LP/DEE techniques to locate the next optimal solution and its corresponding motif.

To identify multiple occurrences of a motif in some of the input sequences, it is possible to iteratively solve several ILPs in order to find multiple near-optimal solutions, corresponding to the best cliques of successively decreasing total weights. At iteration *t*, we add *t *- 1 constraints to the ILP formulation so as to exclude all previously discovered solutions:

∑u∈Skxu≤N−1for k=1,...,t−1,     (10)
 MathType@MTEF@5@5@+=feaafiart1ev1aaatCvAUfKttLearuWrP9MDH5MBPbIqV92AaeXatLxBI9gBaebbnrfifHhDYfgasaacH8akY=wiFfYdH8Gipec8Eeeu0xXdbba9frFj0=OqFfea0dXdd9vqai=hGuQ8kuc9pgc9s8qqaq=dirpe0xb9q8qiLsFr0=vr0=vr0dc8meaabaqaciaacaGaaeqabaqabeGadaaakeaafaqabeqacaaabaWaaabuaeaacqWG4baEdaWgaaWcbaGaemyDauhabeaakiabgsMiJkabd6eaojabgkHiTiabigdaXaWcbaGaemyDauNaeyicI4Saem4uam1aaSbaaWqaaiabdUgaRbqabaaaleqaniabggHiLdaakeaacqqGMbGzcqqGVbWBcqqGYbGCcqqGGaaicqWGRbWAcqGH9aqpcqaIXaqmcqGGSaalcqGGUaGlcqGGUaGlcqGGUaGlcqGGSaalcqWG0baDcqGHsislcqaIXaqmcqGGSaalaaGaaCzcaiaaxMaadaqadiqaaiabigdaXiabicdaWaGaayjkaiaawMcaaaaa@5202@

where *S*_*k *_contains the optimal set of vertices found in iteration *k*. This requires that the new solution differs from all previous ones in at least one graph part. We note that to use this type of constraint for the basic formulation of the motif finding problem, the DEE methods given above have to be modified so as not to eliminate nodes taking part in near-optimal but not necessarily optimal solutions. For the subtle motifs problem, existing DEE methods (e.g., [17]) only eliminate nodes and edges based on whether they can take part in any clique in the graph, and thus constraint 10 can be immediately applied to iteratively find cliques of successively decreasing weight.

## Experimental results

We apply our LP/DEE approach to several motif finding problems. We attempt to discover motifs in instances arising from both DNA and protein sequence data, and compare them with known motifs and those found by other motif finding methods. We then consider the phylogenetic footprinting problem, and demonstrate the discovery of multiple motifs.

### Protein motif finding

We study the performance of LP/DEE on a number of protein datasets with different characteristics (summarized in Table 1). The datasets are constructed from SwissProt [29], using the descriptions of [15] for the first two datasets, [36] for the next two, and [43] for the last one. These datasets are highly variable in the number and length of their protein sequences, as well as in the degree of motif conservation. The motif length parameters are set based on the lengths described by the above authors, and the BLOSUM62 substitution matrix is used for all reported results.

**Table 1:** Descriptions of protein datasets. # *Seq*. gives the number of input protein sequences. *Length *gives the length of the protein motif searched for. |*V*| gives the number of vertices in the original graph constructed from the dataset. *DEE *gives the methods used to prune the graph, and are denoted by (1) *clique-bounds *DEE, (2) tighter constrained bounds and (3) *graph decomposition*. |*V*_*DEE*_| is the number of vertices in the graph after pruning. *E-value *lists the e-value of the motif found by the LP/DEE algorithm.

**Dataset**	**# Seq**.	**Length**	**|*V*|**	**DEE**	**|*V*_DEE_|**	**E-value**
Lipocalin	5	16	844	(1)	5	3.80 × 10^-16^
Helix-Turn-Helix	30	20	6870	(1,2,3)	260	3.88 × 10^-67^
Tumor Necrosis Factor	10	17	2329	(1)	10	1.50 × 10^-40^
Zinc Metallopeptidase	10	12	7761	(1,2)	10	5.82 × 10^-23^
Immunoglobulin Fold	18	10	7498	(1,2,3)	187	3.04 × 10^-24^

For each of the test protein datasets, our approach uncovers the optimal solution according to the SP-measure. These discovered motifs correspond to those reported by [15,36,43], and their SP-scores are highly significant, with e-values less than 10^-15 ^for all of them. As described by [15], the HTH dataset is very diverse, and the detection of the motif is a difficult task. Nonetheless, our HTH motif is identical to that of [15], and agrees with the known annotations in every sequence. We likewise find the lipocalin motif; it is a weak motif with few generally conserved residues that is in perfect correspondence with the known lipocalin signature. We also precisely recover the immunoglobulin fold, TNF and zinc metallopeptidase motifs. The protein datasets demonstrate the strength of our graph pruning techniques. The five datasets are of varying difficulty to solve, with some employing the basic *clique-bounds *DEE technique to prune the graphs, while others requiring more elaborate pruning that is constrained by three-way alignments (see Table 1). In each case, the size of the reduced graph is at least an order of magnitude smaller. For three of the five datasets, the pruning procedures alone are able to identify the underlying motifs.

In contrast to [36], who limit sequence lengths to 500, we retain the original protein sequences, making the problem more difficult computationally. For example, the average sequence length in the zinc metallopeptidase dataset is approximately 800, and some sequences are as long as 1300 residues. The motif we recover is identical to the motif reported by [36] in nine of ten sequences (see Additional Table 1); yet, with the difference in the last sequence, the motif discovered by our method is superior both in terms of sequence conservation and statistical significance (with an e-value of 5.7729 × 10^-23 ^for us vs 1.12155 × 10^-21 ^for [36]).

### Detecting bacterial regulatory elements

We apply our method to identify the binding sites of 36 *E.coli *regulatory proteins. We construct our dataset from that of [6,28], as described in [32]. For each binding site, we locate it within the genome and extract up to 600 bp of DNA sequence upstream from the gene it regulates. We remove binding sites for sigma factors, binding sites for transcription factors with fewer than three known sites, and those that could not be unambiguously located in the genome. Motif length parameters are set as reported by [28], except for *crp*, where a length of 18 instead of 22 is used. Background nucleotide frequencies are computed using the upstream regions for each dataset individually. The final dataset consists of 36 transcription factors, each regulating between 3 and 33 genes, with binding site length ranging between 11 and 48 (see Table 2).

**Table 2:** Listing of the transcription factors' datasets (columns 1, 2, and 3) and the results of motif finding by LP/DEE. *TF *is the transcription factor dataset. *Seq *is the number of input sequences. *Len *is the length of the motif searched for. The rest of the listed measures refer to the motifs discovered by the LP/DEE algorithm: *IC *is the average per-column information content [44]; *RE *is the average per-column relative entropy; *E-value *is the e-value, computed according to our statistical significance assessment; *nPC *is the nucleotide level performance coefficient; and *sSn *is the site level sensitivity. The four starred entries indicate potentially non-optimal solutions; entries marked with † indicated usage of the ILP solver.

**TF**	**Seq**	**Len**	**IC**	**RE**	**E-value**	**nPC**	**sSn**
ada	3	31	1.3000	1.0846	9.16 × 10^-1^	0.1341	0.33
araC	4	48	1.1437	0.9940	1.15 × 10^-3^	0.3474	0.50
arcA	11	15	1.2505	1.1992	4.31 × 10^-6^	0.4224	0.73
argR	8	18	1.2990	1.2149	1.30 × 10^-7^	0.2857	0.50
cpxR	7	15	1.3290	1.2337	1.09 × 10^-5^	0.5556	0.71
crp*†	33	18	0.7196	0.7045	3.08 × 10^-9^	0.5570	0.76
cytR	5	18	1.2317	1.1069	2.48 × 10^-1^	0.0588	0.20
dnaA	6	15	1.4535	1.3300	6.12 × 10^-6^	1.0000	1.00
fadR	5	17	1.3466	1.2074	1.33 × 10^-2^	0.5455	0.80
fis*	8	35	0.8927	0.8376	1.37 × 10^-6^	0.1966	0.38
flhCD	3	31	1.3942	1.1656	4.79 × 10^-3^	0.0000	0.00
fnr	10	22	1.1025	1.0476	1.85 × 10^-9^	0.6176	0.80
fruR	10	16	1.2094	1.1491	5.52 × 10^-8^	0.8182	0.90
fur	7	18	1.3285	1.2332	1.28 × 10^-8^	0.4237	0.71
galR	7	16	1.5445	1.4347	1.52 × 10^-16^	0.5034	0.71
glpR	4	20	1.4227	1.2441	2.63 × 10^-2^	0.5534	0.75
hns	5	11	1.5175	1.3660	2.25	0.0000	0.00
ihf*	19	48	0.3932	0.3859	2.26 × 10^+8^	0.0381	0.16
lexA	17	20	1.1481	1.1192	1.01 × 10^-40^	0.7215	0.88
lrp	4	25	1.2879	1.1237	6.44 × 10^-2^	0.0989	0.25
malT	6	10	1.5071	1.3815	1.73 × 10^-1^	0.0000	0.00
metJ	5	16	1.6842	1.5195	3.37 × 10^-12^	0.6495	1.00
metR	6	15	1.3097	1.1970	6.57 × 10^-2^	0.0000	0.00
modE	3	24	1.5618	1.3145	3.95 × 10^-4^	1.0000	1.00
nagC	5	23	1.2795	1.1462	1.03 × 10^-3^	0.0360	0.20
narL	10	16	1.1391	1.0828	8.06 × 10^-4^	0.8182	0.90
narP	4	16	1.4534	1.2737	7.48 × 10^-4^	0.0000	0.00
ntrC	4	17	1.6621	1.4605	1.28 × 10^-8^	0.6386	1.00
ompR	4	20	1.3566	1.1860	4.27 × 10^-6^	0.0000	0.00
oxyR	4	39	1.0965	0.9521	2.64	0.0796	0.25
phoB	8	22	1.1567	1.0835	4.14 × 10^-9^	0.8051	1.00
purR	20	26	0.8305	0.8147	1.53 × 10^-37^	0.7247	0.95
soxS*†	11	35	0.7771	0.7453	1.26 × 10^-9^	0.0815	0.27
trpR	4	24	1.4069	1.2291	3.74 × 10^-6^	0.8462	1.00
tus	5	23	1.5839	1.4276	1.05 × 10^-17^	0.8400	1.00
tyrR	10	22	1.0693	1.0159	3.63 × 10^-9^	0.5017	0.70

We evaluate the overlap between motif predictions made by our approach and the known motifs using the nucleotide level *performance coefficient *(*nPC*) [1,17]. Let *nTP*, *nFP*, *nTN*, *nFN *refer to nucleotide level true positives, false positives, true negatives and false negatives respectively. For example, *nTP *is the number of nucleotides in common between the known and predicted motifs. The *nPC *is defined as *nTP*/(*nTP *+ *nFN *+ *nFP*); it is a stringent statistic, penalizing a method for both failing to identify any nucleotide belonging to the motif as well as falsely predicting any nucleotide not belonging to the motif. Though *nPC *takes both false positives and false negatives at the nucleotide level into account, we also find it useful to consider site level statistics. Following [1], we consider two sites to be overlapping if they overlap by at least one-quarter the length of the site. Defining site level statistics similarly to the nucleotide level statistics above (e.g., site level true positives, *sTP*, is the number of known sites overlapped by predicted sites), site level sensitivity *sSn *is *sTP*/(*sTP *+ *sFN*).

### Motif finding in genomic data

We compare the performance of our method to three others, MEME [18], Gibbs Motif Sampler [39], and Projection [22]. We choose these particular methods as they are widely-used, readily accessibly for download via the internet, and can handle the lengths of motifs (11–48 bps) in our dataset. While a recent performance evaluation of motif finders [1] indicates that combinatorial methods such as Weeder [24] have somewhat better performance than other methods, most enumerative combinatorial methods, Weeder included, are not able to handle the lengths of the motifs in our bacterial dataset.

Two of the methods we compare against, MEME and Gibbs Motif Sampler, are stochastic-search based algorithms. We run them requiring one motif instance per sequence and limiting the search to the primary sequence strand only, while leaving other parameters at their defaults. Gibbs Motif Sampler is run with 100 random restarts to allow for sufficient sampling of the search space, and MEME is allowed to execute its own algorithm to search the dataset for good starting points for EM. Note that Gibbs Motif Sampler failed to execute on three largest datasets, *crp*, *ihf *and *purr *when run on our local linux machines; these datasets were submitted through the web server.

Projection method has a combinatorial component, combining the idea of locality-sensitive hashing with postprocessing by MEME, and unlike most enumerative combinatorial methods, Projection is not limited by motif length. Since many of the motifs in our dataset are not well conserved, we set the *d *parameter, assessing the average number of mismatches per motif instance, to it maximum suggested value of 0.25 of the motif length. Length parameters of the known motif are used for each dataset.

We detail the performance of our algorithm on the full set of binding sites for 36 *E. coli *transcription factors in Table 2. Considering a motif to have been correctly identified if at least half of its sites were found with at least 25% overlap with the known site (as in [1], and essentially corresponding to datasets with *sSn *of at least 0.5), our method accurately discovers motifs for 22 transcription factors. Setting an e-value threshold at 1.0 (a lower threshold causes other methods to identify too few datasets as having significant motifs), we find statistically significant motifs for 33 datasets. Of the three transcription factor datasets with no significant solutions, one, *hns*, is a short motif, and the other two, *oxyr *and *ihf*, are very poorly conserved motifs with low information content (IC) [44], such that the average per-column IC for the known *oxyr *and *ihf *motifs are 0.89 and 0.36, respectively, whereas these values are 0.95 and 0.39 for their discovered motifs. In general, as compared to the motifs corresponding to the actual known transcription factor binding sites, the motifs found by our method exhibit equal or higher IC, measuring motif conservation in isolation from background sequence, as well as higher relative entropy, measuring the difference with the background distribution (Table 2).

MEME reports motifs for all 36 transcription factor datasets, with e-values less than 1.0 for 20 of them. Gibbs Motif Sampler discovers motifs for 34 of the transcription factors, with 15 of them considered significant via their positive *logMAP *scores (no motifs are found for *araC *and *flhCD*). Projection reports motifs with no significance assessment for all 36 transcription factor datasets. The LP/DEE approach described in this paper has the best overall performance. Taking significance assessment into account, and considering all datasets with no significant motifs to have zero *sSn *and *nPC *values, our method produces 0.554 averaged *sSn *and 0.411 *nPC *values. Indeed, only two datasets, *oxyr *and *ihf*, have motifs that are deemed insignificant using our scheme yet have non-zero overlap with the actual motifs. Performance statistics for MEME and Gibbs Motif Sampler are considerably lower with the averaged *sSn *of 0.338 and *nPC *of 0.257 for Gibbs, and corresponding *sSn *and *nPC *values of 0.382 and 0.285 for MEME. Since Projection does not report significance values, we also note averages of raw coefficients for overlap with the known motifs while ignoring significance assessments. Our method still outperforms the others, though not as significantly, with *sSn *and *nPC *values of 0.565 and 0.414 for LP/DEE; 0.550 and 0.402 for Gibbs; 0.501 and 0.358 for MEME; 0.560 and 0.407 for Projection.

**Figure 1:**
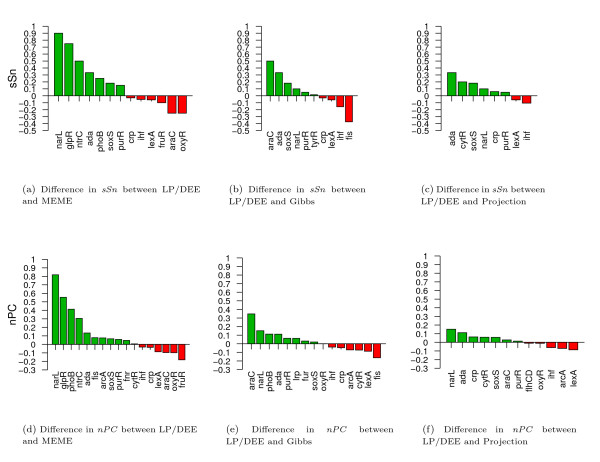
**Performance comparison for the LP/DEE method with Gibbs Motif Sampler, MEME and Projection**. Performance comparison of the LP/DEE method with Gibbs Motif Sampler, MEME, and Projection when identifying *E. coli *regulatory sites. Performance is given in terms of the site level sensitivity 1(a)-1(c) and nucleotide performance coefficient 1(d)-1(f). Significance assessment is disregarded. For every transcription factor dataset, the height of the bar indicates the difference in the metric, with bars above zero specifying better performance for LP/DEE and bars below zero otherwise. Plotted are only those datasets for which there is a difference in performance between the pair of methods being compared.

We also show *sSn *and *nPC *values while ignoring significance for each of the three other methods compared to LP/DEE in Figure 1, only displaying transcription factor datasets for which a difference in performance is observed. Each bar in the chart measures the difference in *sSn *(Figures 1(a)–1(c)) or *nPC *(Figures 1(d)–1(f)) between our method and one of the other methods. Using both the *sSn *and the *nPC *statistics, LP/DEE performs better than any of the three other approaches in identifying known binding sites. For example, for LP/DEE versus MEME, very large differences are observed for three transcription factors, with our method identifying *narL*, *glpR*, and *ntrC *motifs almost completely, and MEME entirely misidentifying them. Moreover, the LP/DEE method exhibits better performance on more transcription factor datasets than the other methods. For example, considering *nPC*, LP/DEE performs better than MEME on eleven datasets, and worse than it on six datasets (Figure 1(e)). Differences in performance with Gibbs Motif Sampler and Projection are smaller; for instance, the LP/DEE method exhibits better performance than Projection using the *sSn *statistic on six datasets versus worse than it on two datasets (Figure 1(c)). We note that if significance assessments are included and motifs with e-value greater than 1.0 are discarded (see Additional Figures 1(a) and 1(b)), then LP/DEE has better *nPC *than MEME on 16 datasets, and worse *nPC *on three datasets, suggesting that MEME's significance computation is unnecessarily conservative for our dataset; the same applies to Gibbs Motif Sampler as well.

Our approach finds provably optimal solutions for 32 of the 36 datasets. Notwithstanding, our method also exhibits excellent runtimes for most problems. Of the 36 transcription factors we considered, 25 were solved in seconds with the application of *clique-bounds DEE*, some using tighter bounds constrained by three-way alignments. Seven required the application of *graph decomposition *with tighter *clique-bounds DEE*, and took a few minutes to three hours to solve. For the remaining four datasets, we used more computationally intensive speculative pruning. Interestingly, we found highly significant solutions for three of these datasets, albeit without the guarantee of optimality, and no significant solution for one. For each of them we provide a bound on the significance value of a potential optimal solution according to the method detailed in the section above. The e-values of the obtained motif and the lower bound in parentheses are: *crp*, 3.08 × 10^-9 ^(6.04 × 10^-33^); *fis*, 1.37 × 10^-6 ^(2.29 × 10^-7^); *soxS*, 1.26 × 10^-9 ^(5.25 × 10^-14^); and *ihf*, 2.26 × 10^+8 ^(3.98 × 10^-31^). Finally, in the entire data collection, all but two of the problems resulted in integral solutions to their LPs. The remaining instances with fractional solutions were easily solved by the ILP solver.

#### Simulated data

We also evaluate the effectiveness of our scoring scheme in finding binding sites for five regulatory proteins when they are embedded in simulated data. Our goals are twofold. First, since our underlying scoring measure is based on counting matches between nucleotides, it is important to see how well it performs in compositionally biased backgrounds. In the *E. coli *dataset, even a simple scoring scheme that assigns a score of 1 to matches and 0 to mismatches performs competitively (data not shown). However, since other genomes can have considerably more biased nucleotide compositions, our scoring scheme rewards matches between more rare nucleotides, and we test here how it performs in different scenarios. Second, while it is essential to test the performance of motif finding algorithms on genomic data (as above), it is possible that there are other conserved motifs in the data, besides those with which we are evaluating performance, and these conserved motifs lead to lower *nPC *and *sSn *measurements. Simulated data is not expected to have other conserved motifs, and thus provides a cleaner, though perhaps optimistic, means for testing motif finding approaches.

In our testing on simulated data, we use a selection of five transcription factor datasets with motifs of varying levels of conservation, as measured by their IC (Table 3). We generate background sequences with uniform nucleotide distributions, as well as those with increasingly biased probability distributions. A background sequence for a particular binding site is generated of length equal to that of its upstream region (up to 600 bps). In particular, for each position, a base is selected at random according to a probability distribution in which base *G *is chosen with some probability *pr*(*G*) and the other bases with probability (1 - *pr*(*G*))/3 each.

**Table 3:** Scoring method evaluation in terms of performance coefficient in biased-composition simulated data. Performance of LP/DEE in biased-composition simulated data. The first column identifies the TF dataset. The second column measures the degree of conservation of the known motif, as measured by average per-column information content [44]. The rest of the columns list the nucleotide performance coefficient of the LP/DEE method with the probability of base *G *indicated in the column heading and the frequencies of all other bases split equally.

**TF**	**IC**	**Bias**			
		
		0.25	0.5	0.75	0.9
araC	1.00041	0.8113	0.9592	0.9592	0.9592
cpxR	1.17034	1.0000	0.8261	0.9811	0.9811
dnaA	1.45351	1.0000	0.7647	0.7647	1.0000
galR	1.34756	0.8824	0.8824	1.0000	1.0000
narP	1.40273	1.0000	1.0000	1.0000	1.0000

Our *nPC *performance is summarized in Table 3 for various background distributions. We find motifs of very high *nPC *values in varying biased nucleotide composition, attesting to the fact that our scoring scheme is successfully able to correct for bias in sequence composition. Moreover, as expected, performance on simulated data is better than that for actual genomic sequence. In the *narP *dataset, for example, the motif is found perfectly in simulated data and not at all in real genomic data. Additionally, an alternate highly conserved site is found by all four methods in genomic data (Table 2), suggesting that while the *narP *site is well-conserved, the corresponding genomic sequences contain another shared motif of higher conservation.

### Phylogenetic footprinting

We also apply our approach to identify motifs among sets of upstream regions of orthologous genes in a number of genomes. Here, the relationships between genomes is incorporated via weighting of the components of the SP-score. The eight datasets come from [9]. All datasets contain vertebrate sequences; some (Interleukin-3 and Insulin datasets) consist of only mammalian genomes, while others contain members from more diverse animal phyla. The number of sequences in the datasets ranges between 4 and 16, and most sequences are shorter than 1000 residues in length.

We use the phylogenetic trees (topology and branch lengths) given in [9] to derive the pairwise weights, and use the motif lengths provided. For each of the eight datasets, our approach identifies the optimal motif using the SP scoring measure (Table 4). The consensus sequences for the discovered motifs are listed in Table 4 along with the description of their DNA regions. (The motif reported for the *c-fos *promoter dataset was discovered second, after having discarded the poly-A repeat region.) All the motifs we find have been documented in the TRANSFAC database [45], and the majority of them correspond to those that have been reported by [9]. Two motifs differ from those of [9]: the first, a *c-fos *motif, shares its consensus sequence with a known *c-fos *regulatory element, the binding site of the serum response factor (SRF) protein (accession number R02246). The second, a *c-myc *motif, also corresponds to a known *c-myc *binding site in the *P1 *promoter (accession number R04621). The e-values of the found motifs range from 10^-18 ^to 10^-5^. We note that though the notion of significance according to our method merely rejects the hypothesis that all the motif instances are unrelated, and a scheme that takes phylogeny into account such as [46] may be better suited for this problem in general, our significance evaluation attests to the presence of a highly conserved motif instance in every input sequence.

**Table 4:** Motifs identified with use of phylogenetic information. Listing of motifs and details of their host sequences for phylogenetic motif finding. All datasets tested are from [9]. *DNA region *details the DNA regions considered (PR signifies promoter region). *# Seq*. gives the number of input sequences. *Motif (id) *identifies the consensus sequence of the discovered motif and its correspondence with the motifs of [9] where applicable. All listed motifs have been documented as regulatory elements in TRANSFAC [45]. For datasets other than the *insulin *dataset, only the best motif is reported and for the *insulin *dataset multiple motifs are reported in order of discovery.

**DNA region**	**# Seq**.	**Motif (id)**
Growth-horm. 5' UTR + PR (380 bp)	16	TATAAAAA (7)
Histone H1 5' UTR + PR (650 bp)	4	AAACAAAAGT (2)
C-fos 5' UTR + PR (800 bp)	6	CCATATTAGG
C-fos first intron (376 to 758 bp)	7	AGGGATATTT (3)
Interleukin-3 5' UTR + PR (490 bp)	6	TGGAGGTTCC (3)
C-myc second intron (971 to 1376 bp)	6	TTTGCAGCTA (5)
C-myc 5' PR (1000 bp)	7	GCCCCTCCCG
Insulin family 5' PR (500 bp)	8	GCCATCTGCC (2) TAAGACTCTA (1) CTATAAAGCC (3) CAGGGAAATG (4)

This dataset is also an excellent testing ground for finding distinct multiple motifs using our method. We iteratively identify motifs and remove their corresponding vertices from the constructed graphs. As proof of principle, we find multiple motifs for the insulin dataset. In this case, we successfully identify all four motifs reported by [9]. Since our objective function differs from theirs and we require motif occurrences in every input sequence, we recover the motifs in a different order. Of course, we identify numerous shifts of these motifs in successive iterations. In practice, therefore, it may be more beneficial to remove a number of vertices corresponding to subsequences overlapping the optimal solution before attempting to find the next motif.

## Conclusion

We have described a combined graph-theoretic and mathematical programming framework for the motif finding problem that provides a flexible approach to tackle many important issues in motif finding. We have successfully applied it to a variety of problems, including discovering statistically significant DNA and protein motifs, and have been able to incorporate phylogenetic information in the context of cross-species motif discovery. A major advantage of our approach for motif finding is the ability to find optimal solutions for many practical problems.

In related follow up work, we have shown how to improve the ILP formulation in the case where there are only a small number of distinct edge weights [47]; while this is not the case with the similarity scores considered here, it comes up in some applications (e.g., when considering scores based on just the total number of exact base-pair or amino acid matches). Further improvements and extensions to the ILP formulation for motif finding may be possible – for example, by incorporating constraints that model cooperative binding of transcription factors by looking for motifs within some distance of one another. While mathematical programming has not traditionally been applied to the motif discovery problem, our work demonstrates that it provides us a powerful alternative to successfully tackle a diverse set of applications.

## Supplementary Material

Additional File 1 1. A table with the sequences for the human zinc metallopeptidase motif as found by the LP/DEE method and [36]. 2. A figure describing performance comparison between the LP/DEE method and MEME when using a significance threshold as reported by both methods. Click here for file
