# Supplementary material for: A combinatorial optimization approach for diverse motif finding applications
Source: Algorithms Mol Biol. 2006 Aug 17;1:13. doi: 10.1186/1748-7188-1-13 (PMC1570465; doi:10.1186/1748-7188-1-13)
Supplement: Additional File 1 — 1. A table with the sequences for the human zinc metallopeptidase motif as found by the LP/DEE method and [36]. 2. A figure describing performance comparison between the LP/DEE method and MEME when using a significance threshold as reported by both methods. [file 1748-7188-1-13-S1.pdf]

Supplementary Material

ID	LP/DEE Motif		Lukashin Motif	
	Position	Motif	Position	Motif
ACET	411	VAHHEMGHIQYF	411	VAHHEMGHIQYF
AMPN	384	VIAHELAHQWFG	384	VIAHELAHQWFG
BMP1	210	IVVHELGHVVGF	210	IVVHELGHVVGF
MM01	215	VAAHELGHSLGL	215	VAAHELGHSLGL
MM02	400	VAAHEFGHAMGL	400	VAAHEFGHAMGL
MM03	215	VAAHEIGHSLGL	215	VAAHEIGHSLGL
LKHA	292	VIAHEISHSWTG	292	VIAHEISHSWTG
MEPA	152	IIEHEILHALGF	152	IIEHEILHALGF
PSA	349	VVGHELAHQWFG	349	VVGHELAHQWFG
ACE	985	VAHHEMGHIQYF	387	TVHHEMGHIQYY

Table 1: Human zinc metallopeptidase motif as found by the LP/DEE method and Lukashin & Rosa [41]. All sequences are designated by their SwissProt entries with extension *HUMAN* omitted.

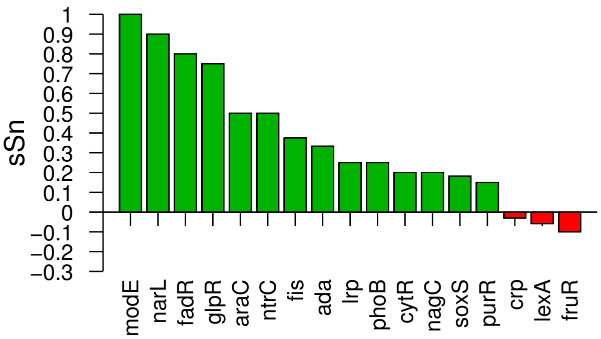

(a) Difference in site sensitivity between LP/DEE and MEME using a significance threshold.

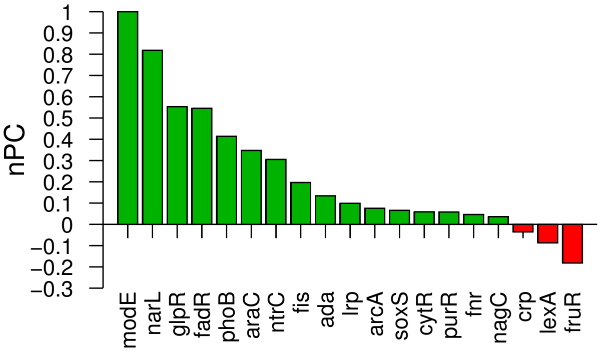

(b) Difference in nucleotide performance coefficient between LP/DEE and MEME using a significance threshold.

Figure 1: Performance comparison between the LP/DEE method and MEME when identifying known regulatory sites when using a significance threshold. For every transcription factor dataset, the height of the bar indicates the difference in the metric, with bars above zero specifying better performance for our method and bars below zero otherwise. When a method fails to report a statistically significant motif with an e-value cutoff of 1.0, the number of correctly identified nucleotides and sites is set to zero.
